# Elevated Adenosine Kinase and Decreased *ENTPD1* mRNA Levels in Peripheral Blood Leukocytes of Patients with MASLD Differentiate Metabolic Dysfunction-Associated Steatohepatitis from Hepatic Steatosis

**DOI:** 10.3390/diseases14100372

**Published:** 2026-10-10

**Authors:** Irina Kurbatova, Galina Zhulai, Olga Dudanova, Lyudmila Topchieva

**Affiliations:** 1 Institute of Biology, Karelian Research Centre of the Russian Academy of Sciences, Petrozavodsk 185910, Republic of Karelia, Russia; zhgali-111@yandex.ru (G.Z.); odudanova@gmail.com (O.D.); topchieva67@mail.ru (L.T.); 2 Department of Propaedeutics of Internal Diseases and Hygiene, Medical Institute Named After Professor A.P. Zilber, Petrozavodsk State University, Petrozavodsk 185910, Republic of Karelia, Russia

**Keywords:** metabolic dysfunction-associated steatotic liver disease (MASLD), metabolic dysfunction-associated steatohepatitis (MASH), adenosine kinase, CD39, CD73, *ENTPD1*, *NT5E*, CD14^+^ monocytes, CD4^+^ T cells

## Abstract

**Background:** Adenosine kinase (ADK) and the ectonucleotidases CD39 and CD73 regulate adenosine metabolism and play important roles in the pathogenesis of liver diseases. Their involvement in metabolic dysfunction-associated steatotic liver disease (MASLD) remains insufficiently characterized, particularly in humans. This study aimed to comparatively analyze ADK levels, the mRNA levels of the long and short *ADK* isoforms and the *ENTPD1*, *NT5E*, and *ADORA2A* genes in peripheral blood leukocytes (PBLs), and the proportion of CD39- and CD73-expressing cells in the blood of healthy individuals and patients with different forms of MASLD (hepatic steatosis (HS) and metabolic dysfunction-associated steatohepatitis (MASH)). **Methods:** Blood samples were obtained from 42 healthy individuals and 98 patients with MASLD. ADK and cytokine levels were measured by ELISA. mRNA levels were determined by RT-PCR. The proportions of CD39- and CD73-expressing cells were determined by flow cytometry. **Results:** Patients with HS and MASH differed in ADK concentration and relative *ENTPD1* gene expression in PBL. Compared with HS, MASH was characterized by higher ADK concentrations and lower *ENTPD1* mRNA levels, both of which showed potential for distinguishing MASH from HS: ADK, *AUC* = 0.764 (95% CI, 0.637–0.892; *p* = 0.001); *ENTPD1* mRNA, *AUC* = 0.649 (95% CI, 0.546–0.752; *p* = 0.007). Patients with MASLD, including those with HS and MASH, had lower proportions of CD39^+^ monocytes than controls, whereas the proportions of CD4^+^CD39^+^ T helper cells did not differ significantly between the groups. **Conclusions:** ADK levels in PBLs may be associated with the presence and activity of inflammation in MASLD. Compared with patients with HS and healthy controls, patients with MASH had elevated ADK levels and lower relative levels of *ENTPD1* transcripts in PBLs. The latter finding may be related to the reduced proportion of CD39^+^ monocytes.

## 1. Introduction

Metabolic dysfunction-associated steatotic liver disease (MASLD) (previously known as non-alcoholic fatty liver disease, NAFLD) is a slowly progressive, extremely common, chronic, non-infectious liver disease. According to recent data, the global prevalence of MASLD exceeds 30% in the adult population [1,2]. MASLD encompasses a spectrum of clinical and morphological forms, including simple hepatic steatosis (HS) and metabolic dysfunction-associated steatohepatitis (MASH) [3]. A quarter of patients with hepatic steatosis develop liver fibrosis [4]; in 15–25% of cases, patients with MASH develop severe fibrosis and cirrhosis, with the possibility of developing hepatocellular carcinoma [5]. In contrast to HS, which is characterized by a benign clinical course, MASH is characterized by lobular inflammation and ballooning degeneration of hepatocytes (with or without fibrosis) and can progress to cirrhosis and hepatocellular carcinoma. It should be noted that the search for minimally invasive alternatives to liver biopsy for differentiating MASH from HS remains a pressing problem in gastroenterology, since liver biopsy has serious limitations and may not reflect the true picture of damage, inflammation, and fibrosis due to the small tissue volume [6].

Along with lipotoxicity, oxidative stress, the development of insulin resistance, impaired autophagy, hepatocyte death, and the development of fibrosis, the complex pathogenesis of MASLD includes inflammation [3,7]. The development of hepatocellular failure in MASLD is associated with parenchymal loss due to leukocyte infiltration and activation of proinflammatory immune cell populations [7]. Circulating CD4^+^ T helper cells and monocytes play a significant role in this process [8]. Monocytes and monocyte-derived macrophages are important sources of cytokines in the liver and play a significant role in the progression and resolution of MASH [9]. There is evidence that, in the liver, particularly in the context of MASH development, a large proportion of macrophages are recruited, including monocyte-derived macrophages [9]. Peripheral monocytes differ in their proinflammatory potential: CD14^hi^CD16^−^ classical monocytes, whose main function is phagocytosis, whereas CD14^+^CD16^+^ intermediate and CD14^lo^CD16^+^ non-classical monocytes promote the development of inflammation [10].

One promising area of inflammation research is the adenosine pathway. At sites of tissue damage, extracellular adenosine accumulates and plays a key role in modulating the immune response and limiting inflammatory tissue damage by activating adenosine receptors on immune cells—primarily A2AR (adenosine A2A receptor), encoded by the *ADORA2A* gene. By regulating various inflammatory immune cells, adenosine protects tissues from damage and stress [11,12]. It is formed from ATP released during necrosis, apoptosis, or hypoxia. Two key enzymes drive this process: ectonucleoside triphosphate diphosphohydrolase 1 (ENTPD1, or CD39), which converts adenosine triphosphate (ATP) and adenosine diphosphate (ADP) to 5′-adenosine monophosphate (AMP), and ecto-5′-nucleotidase (NT5E, or CD73), which then generates adenosine from AMP [13]. Growing evidence points to the adenosine pathway as an important player in liver diseases [14,15,16,17]. Through dephosphorylation of ATP to adenosine, CD39 and CD73 exert protective effects against local and systemic inflammation [18,19,20,21,22]. Notably, however, CD73 also displays profibrotic activity [23,24].

Among components of the adenosine pathway in liver disease, adenosine kinase (ADK) is especially noteworthy. This ribokinase-family enzyme eliminates adenosine by phosphorylating it to AMP [25]. Through this action, it controls adenosine availability and serves as a key node in homeostatic and metabolic networks. It also sustains normal adenine nucleotide levels and supports S-adenosylmethionine-dependent transmethylation, a process in which the obligate byproduct adenosine must be continually cleared. In healthy cells, the amounts of adenosine and ADK are tightly balanced. Altered expression of the enzyme changes the degree of adenosine receptor activation and thereby often shapes its pathogenic role [26]. The ADK enzyme exists in two isoforms formed as a result of alternative splicing [27], which are similar in biochemical and kinetic properties. They differ structurally only at the N-terminus: the long isoform (ADK-L) has an additional 20–21 amino acids, while the short isoform (ADK-S) has four. Their intracellular distributions also diverge—ADK-L is nuclear, whereas ADK-S is cytoplasmic [28]—which suggests functional specialization. The short isoform phosphorylates adenosine and thus governs how much is available for receptor activation, whereas the long one acts as an epigenetic regulator in transmethylation reactions [29].

ADK is present in most tissues, with the highest amounts in the liver [30,31]. Genetic disruption of ADK expression results in severe liver dysfunction. Homozygous Adk^−/−^ mutant mice develop normally during embryogenesis, but within four days of birth they exhibit microvesicular hepatic steatosis and die within two weeks of fatty liver disease [30]. The livers of these mice were characterized by reduced ATP stores, accumulation of S-adenosylhomocysteine, an inhibitor of transmethylation, and accumulation of adenosine. Human ADK deficiency, first described in 2011, is one of the inborn errors of metabolism at the junction of methionine and adenosine metabolism: three homozygous missense mutations of the *ADK* gene (c.902C>A (p.Ala301Glu), c.653A>C (p.Asp218Ala), and c.38G>A (p.Gly13Glu)) lead to a severe metabolic disorder, which is accompanied by increased levels of methionine, S-adenosylmethionine, and S-adenosylhomocysteine, developmental delay, and impaired liver function [31,32,33]. The dominant histological feature in these patients is severe microvesicular hepatic steatosis, along with liver dysfunction that occurs in the neonatal period and hyperinsulinemic hypoglycemia [31,32].

Some studies indicate that ADK plays a significant role in the processes associated with the pathogenesis of MASLD: lipotoxicity, insulin resistance, and inflammation [34,35,36]. Hepatic ADK levels have been shown to be elevated in MASLD patients and in mice with diet-induced obesity, hepatic steatosis, and insulin resistance. Experiments with *ADK* gene knockout and overexpression in hepatocytes of experimental models have shown that ADK in hepatocytes promotes excessive fat accumulation and inflammation in the liver by suppressing fatty acid oxidation in hepatocytes and promoting the production of proinflammatory mediators [35]. Another study showed that a high-fat diet (HFD) increases ADK expression in the endothelium. Endothelial *Adk* deficiency protects mice from HFD-induced insulin resistance, improves glucose tolerance and insulin sensitivity, enhances NO production, and reduces liver steatosis and adipose tissue inflammation by increasing intracellular adenosine [34]. ADK ablation in pancreatic β cells improves glucose tolerance and β-cell function in mice [36].

Based on the above, it can be concluded that regulators of adenosine metabolism (ectonucleotidases CD39 and CD73, involved in the degradation of ATP and AMP to adenosine, and adenosine kinase, which phosphorylates adenosine to AMP) play an important role in the pathogenesis of liver diseases. However, information on their involvement in the pathogenesis of MASLD is insufficient and has been obtained mainly from experimental models. Data on the relationship between the level of ADK and the CD39–CD73–A2AR signaling pathway and inflammation in liver dysfunction in MASLD are lacking. The progression of hepatocellular failure in MASLD is directly related to parenchymal loss due to leukocyte infiltration and the initiation of immunoinflammatory processes [7]. Considering the anti-inflammatory role of CD39 [18,20,21], CD73 [22], and A2AR [16] and the proinflammatory role of ADK [34,35] in the liver, it can be expected that the levels of these molecules in the peripheral blood may be associated with the presence and activity of inflammation in the liver parenchyma in MASLD and may differ depending on the form of the disease. In this regard, the aim of the study was a comparative analysis of the levels of ADK, mRNA of the *ADK* (long and short isoforms), *ENTPD1*, *NT5E*, and *ADORA2A* genes in peripheral blood leukocytes (PBLs) and the content of CD39- and CD73-expressing cells in the blood of healthy individuals and patients with different forms of MASLD (HS, MASH), as well as an assessment of the correlations between ADK content and mRNA levels of the spliced forms of the *ADK* gene in PBLs and routine clinical parameters, the levels of TNFα, IL-6, IL-1β, and IL-10 cytokines in blood plasma, and the content of CD39- and CD73-expressing CD4^+^ T helper cells (Th) and CD14^+^ monocytes in the blood of healthy individuals and patients with different forms of MASLD (HS, MASH).

## 2. Materials and Methods

### 2.1. Patients and Controls

The study included 42 healthy individuals (control group) and 98 patients with MASLD: 50 individuals with simple hepatic steatosis (HS group) and 48 individuals with metabolic dysfunction-associated steatohepatitis (MASH group). Informed consent was obtained from all subjects involved in the study. Healthy donors and MASLD patients were examined by gastroenterologists at the Russian Railways Medicine Clinical Hospital (Petrozavodsk, Russia). The clinical and laboratory characteristics of the groups are presented in Table 1.

MASLD was diagnosed using conventional clinical, laboratory, instrumental, and histological data. All patients displayed features of metabolic syndrome: body mass index (BMI) > 30 kg/m^2^; waist circumference > 102 cm in men and > 88 cm in women; dyslipidemia, indicated by elevated low-density lipoprotein (LDL) > 3.0 mmol/L; systolic blood pressure ≥ 130 mmHg; and diastolic blood pressure ≥ 80 mmHg. Abdominal ultrasonography (Vivid Pro-7, General Electric, Milwaukee, Wisconsin, USA) assessed liver echogenicity and dimensions, splenic size, portal and splenic vein diameters, and Doppler-derived linear flow velocity in these vessels. No subject showed portal hypertension: ascites was absent, and esophagogastroscopy found neither esophageal nor gastric cardiac varices. Spiral computed tomography of the liver, including density measurement, was performed. Hepatic stiffness was assessed by ARFI (Acoustic Radiation Force Impulse) shear wave elastography using a Mindray DC-80 (Mindray, Shenzhen, China). Patients also underwent blind percutaneous liver biopsy with evaluation of histological activity and fibrosis according to the Brunt et al. method [37]; this verified the MASLD form: steatosis (≥5% hepatocytes) without inflammation and ballooning of hepatocytes, or steatohepatitis with active injury—hepatocellular ballooning degeneration and lobular inflammation (mainly lymphocytic, with some neutrophils)—alongside varying degrees of steatosis. Among MASH patients, there were no individuals with advanced fibrosis (histologically, it did not exceed F2, and elastographic stiffness was ≤9.5 kPa).

Shared exclusion criteria were infectious or inflammatory disease within the past month, pregnancy or lactation, and smoking. In the examined patients, the following liver diseases were ruled out: viral disease by negative HBV/HCV serology; autoimmune disease by absence of autoantibodies to hepatocyte structures; alcohol-related disease by medical history, clinical findings, and CAGE/AUDIT scores; and drug-induced disease by history alone. No participant had type 1 or type 2 diabetes mellitus. Healthy donors lacked cardiometabolic risk factors and had normal BMI, liver function tests, and hepatic sonographic structure and stiffness.

The study complied with the Declaration of Helsinki and was approved by the Medical Ethics Committee of the Ministry of Healthcare of the Republic of Karelia and of Petrozavodsk State University (protocol no. 48, 10 March 2023).

### 2.2. Blood Sample Collection

Venous blood samples were collected from the cubital vein in fasting patients using tubes containing the anticoagulant K_2_EDTA. Samples were collected from patients during hospitalization before therapy was started, 2–3 days before liver biopsy.

### 2.3. Analysis of Clinical and Laboratory Blood Parameters

Liver function parameters—alanine aminotransferase (ALT), aspartate aminotransferase (AST), total bilirubin, alkaline phosphatase (ALP), gamma-glutamyl transpeptidase (GGT), total protein, glucose, total cholesterol (TC), high-density lipoprotein (HDL), triglycerides, and C-reactive protein (CRP)—were measured using the MIURA analyzer (I.S.E. S.r.l., Guidonia, Roma, Italy) with reagents from Vector-Best (Novosibirsk, Russia). Low-density lipoprotein (LDL) levels were calculated using the Friedewald formula. The erythrocyte sedimentation rate (ESR) was determined by the Westergren method. The hemogram was evaluated using the MINDRAY BC-6200 (Shenzhen Mindray Bio-Medical Electronics Co., Ltd., Shenzhen, China).

### 2.4. Calculation of FIB-4 Fibrosis Index

FIB-4 was calculated using the formula presented in the work of Sterling et al. [38]. A FIB-4 index value ≥ 2.67 indicates a high risk of fibrosis progression with 80% confidence; a FIB-4 index ≤ 1.30 indicates a low risk with 90% confidence [39].

### 2.5. Measurement of Cytokine Levels in Blood Plasma

The concentrations of proinflammatory (TNFα, IL-6, and IL-1β) and anti-inflammatory (IL-10) cytokines in blood plasma were determined by ELISA using a SuPerMax 3100 multimodal plate reader (Flash Spectrum, Shanghai, China). The test systems Alpha–TNF–ELISA–Best, Interleukin-6–ELISA–Best, Interleukin-1 Beta–ELISA–Best, and Interleukin-10–ELISA–Best (Vector-Best, Novosibirsk, Russia) were used in accordance with the manufacturer’s instructions.

### 2.6. Measurement of ADK Concentration in Peripheral Blood Leukocytes

To determine ADK concentration, PBLs were isolated. Fasting venous blood was treated with a lysis solution containing 114 mM NH_4_Cl, 7.5 mM KHCO_3_, and 100 μM EDTA for 10 min. After centrifugation at 1250 rpm for 7 min, the leukocyte fraction was collected. This procedure was repeated twice. PBLs were lysed using freeze–thaw cycles. ADK concentration in PBLs was determined by ELISA using the Adenosine Kinase ELISA Kit (Cloud-Clone Corp., Katy, TX, USA) with normalization to the number of viable cells in a given sample volume. The obtained concentration of ADK was expressed in ng/mL, assuming that 1 mL contains 10 million living cells.

### 2.7. RNA Isolation and Gene Expression Analysis

Total RNA from the PBL fraction was isolated using the ExtractRNA reagent (Eurogen, Moscow, Russia). After determining its quality (electropherogram analysis in 1% agarose gel) and quantity (based on the ratio of optical densities at 260 and 280 nm), the total RNA was treated with DNase E (Eurogen, Moscow, Russia) according to the manufacturer’s instructions. The first strand of complementary DNA (cDNA) was synthesized from total RNA using the MMLV RT kit (Eurogen, Moscow, Russia). Purity and cDNA concentration were determined using the SmartSpecPlus instrument (Bio-Rad, Hercules, CA, USA). The relative level of gene transcripts in PBLs was assessed by RT-PCR (real-time PCR) on the Light Cycler (Roche, Mannheim, Germany) using the qPCRmix-HS SYBR kit (Eurogen, Moscow, Russia). Primers for amplification of the *ADK-L* gene were as follows: forward 5′-TGTAGAGCCAAAGTGGGGTG-3′, reverse 5′-GCCTCCACCTTCAGCTTTTTG-3′; the *ADK-S* gene: forward 5′-AAGCAGTTGCTGTGGTACCTG-3′, reverse 5′-AGCAGAGGATTTCCCATTCCA-3′; the *ADORA2A* gene: forward 5′-CTTGGGTTCTGAGGAAGCAG-3′, reverse 5′-CAGCAGCTCCTGAACCCTAG-3′; *ENTPD1* gene: forward 5′-AGCAGCTGAAATATGCTGGC-3′, reverse 5′-GAGACAGTATCTGCCGAAGTCC-3′; *NT5E* gene: forward 5′-ATTGCAAAGTGGTTCAAAGTCA-3′, reverse 5′-ACACTTGGCCAGTAAAATAGGG-3′. The *18S rRNA* gene was used as a reference gene, the primers for which were as follows: forward 5′-AGAAACGGCTACCACATCCA-3′, reverse 5′-CACCAGACTTGCCCTCCA-3′. The PCR conditions were as follows: cDNA denaturation for 5 min at 95 °C; 40 cycles: denaturation at 95 °C for 30 s, annealing at the temperature corresponding to the primers for 30 s, and elongation at 72 °C for 30 s. PCR efficiency was assessed using a standard curve, and the specificity of PCR products was assessed using melting curves. PCR analysis was repeated three times. Relative transcript levels were estimated according to Livak and Schmittgen [40].

### 2.8. Flow Cytometry

For flow cytometry analysis, peripheral blood samples from 22 healthy donors (control), 43 patients with HS, and 30 patients with MASH were examined. The blood samples were stained with CD39-FITC, CD73-PE, CD3-PE-Cy5, CD4-PE-Cy7, CD16-PE-Cy5, and CD14-PE-Cy7 monoclonal antibodies, as well as appropriate isotype controls (Sony Biotechnology, San Jose, CA, USA) for 20 min. Red blood cells were lysed with BD FACS Lysing Solution (BD Biosciences, San Jose, CA, USA). A lymphocyte and monocyte region was identified based on forward scatter (FS) and side scatter (SS). Subsequently, T-helper cells were identified based on the expression of CD3 and CD4 markers. Within this population, the relative frequency of CD39- and CD73-expressing cells was analyzed using isotype controls. Monocytes were gated based on CD14 expression, and subpopulations of classical (CD14^hi^CD16^−^), intermediate (CD14^+^CD16^+^), and non-classical (CD14^lo^CD16^+^) monocytes were subsequently identified. The frequency of CD39- and CD73-expressing cells within specific monocyte subpopulations was analyzed using fluorescence minus one (FMO) controls. Data acquisition was performed on a Cytomics FC500 (Beckman Coulter, Miami, Florida, USA). At least 100,000 total events were recorded for each sample. Absolute cell counts were calculated based on clinical blood count data.

### 2.9. Statistical Analysis

Statistical analysis was performed using GraphPad Prism v.8 (GraphPad Software Inc., San Diego, CA, USA). Values of *p* < 0.05 were considered statistically significant. Results were tested for normal distribution using the Shapiro–Wilk *W* test. Due to non-normal distributions of the parameters in the groups, nonparametric tests were used for analysis. To compare the parameters under study between groups, the Kruskal–Wallis *H* test was used, followed by pairwise comparisons using the Mann–Whitney *U* test. Interrelationships between parameters were assessed using the Spearman rank correlation coefficient (*r*). The Chaddock scale was used to assess the strength of the relationship between two variables. Receiver operating characteristic (ROC) analysis was performed by calculating the area under the curve (*AUC*) values; the optimal cutoff threshold was determined in accordance with the maximum Youden index [41,42].

This study was conducted using equipment from the Core Facility of the Karelian Research Centre of the Russian Academy of Sciences.

## 3. Results

### 3.1. Clinical and Laboratory Blood Parameters of Healthy Individuals and Patients with MASLD

The results of the analysis of routine clinical and laboratory blood parameters in healthy individuals and patients with MASLD are presented in Table 1. HS patients had higher ALT, GGT, LDL, and triglyceride levels and lower HDL than controls; other parameters were comparable. The MASLD group also showed higher BMI, and FIB-4 was elevated in MASH versus controls. Histological, BMI, and FIB-4 data revealed no severe fibrosis or morbid obesity among MASLD patients. Relative to HS patients and controls, those with MASH had increased cytolytic markers (ALT, AST), total cholesterol, and LDL, alongside reduced HDL. MASH patients also had higher ALP, GGT, CRP, and triglycerides than controls (Table 1).

### 3.2. The Level of ADK and Splice Isoforms of the ADK Gene in the Peripheral Blood of Healthy Individuals and Patients with MASLD

**Figure 1:**
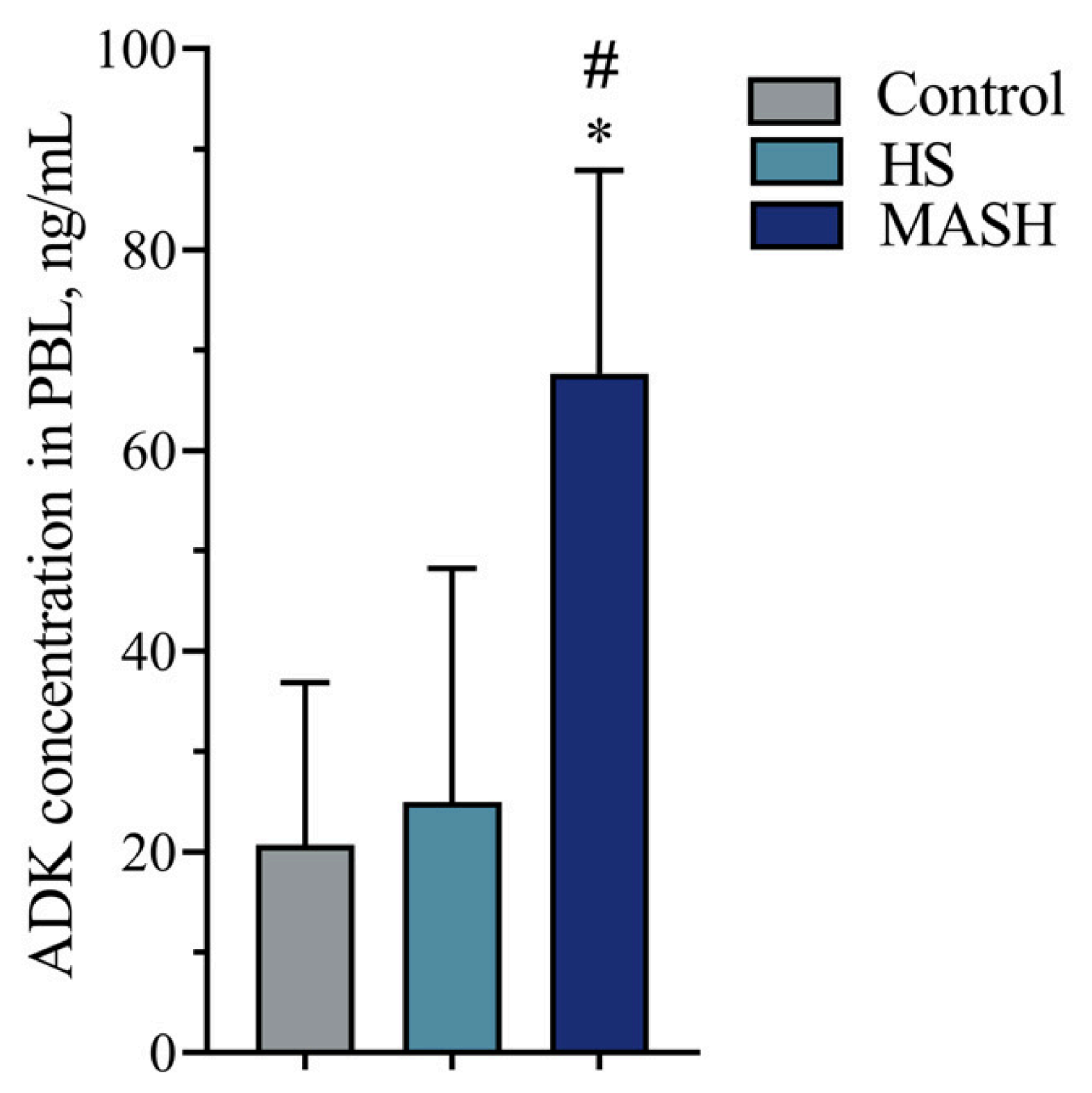
ADK concentration in PBLs of patients with MASLD and healthy donors. *—Significant difference from the control group; #—significant difference from the HS group (according to the Mann–Whitney *U* test with Bonferroni correction). Medians and interquartile ranges are presented. ADK—adenosine kinase; PBL—peripheral blood leukocytes; HS—hepatic steatosis; MASH—metabolic dysfunction-associated steatohepatitis.

According to the Kruskal–Wallis analysis of variance, the study groups (control, HS, and MASH) differed in the level of ADK in PBLs (*H* = 13.610, *p* = 0.001). The concentration of ADK in the PBLs of MASH patients was higher than that in patients with simple hepatic steatosis and healthy individuals (Figure 1).

**Figure 2:**
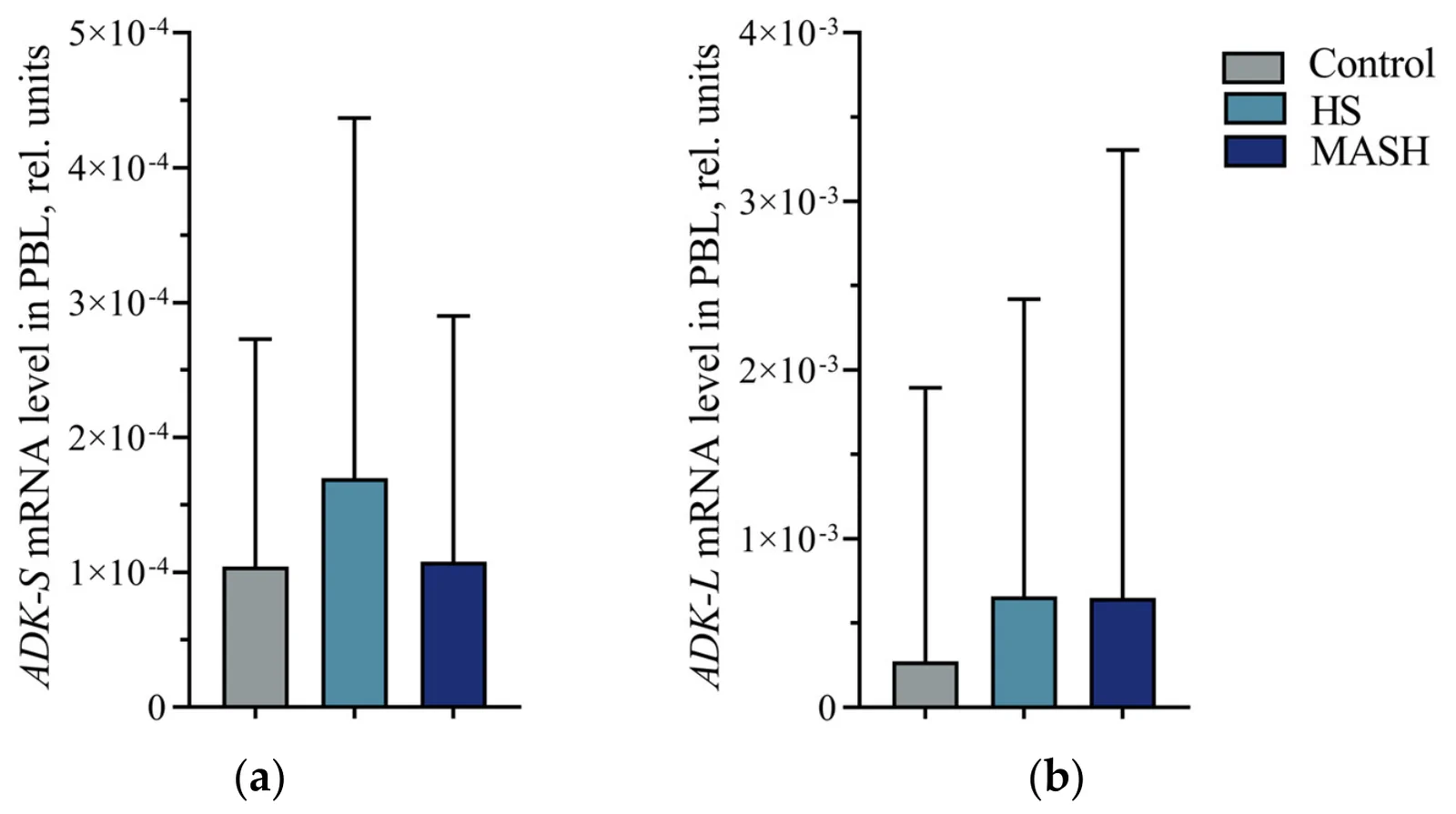
(**a**) mRNA levels of the short isoform of the *ADK* gene (*ADK-S*) in PBLs of patients with MASLD and healthy donors. (**b**) mRNA levels of the long isoform of the *ADK* gene (*ADK-L*) in PBLs of patients with MASLD and healthy donors. Medians and interquartile ranges are presented. *ADK-S*—gene encoding the short isoform of adenosine kinase; *ADK-L*—gene encoding the long isoform of adenosine kinase; PBL—peripheral blood leukocytes; HS—hepatic steatosis; MASH—metabolic dysfunction-associated steatohepatitis.

The study groups did not differ in the mRNA levels of the short and long isoforms of the *ADK* gene in PBLs (*H* = 2.193, *p* = 0.334; *H* = 1.523, *p* = 0.467, respectively) (Figure 2).

The median ratio of the mRNA level of *ADK-L* to the level of *ADK-S* (*ADK-L*/*ADK-S*) in PBLs was 9.665 in the control group, 8.280 in the HS group, and 6.860 in the MASH group. No intergroup differences were found in the ratio of *ADK-L*/*ADK-S* transcript levels (*H* = 0.173, *p* = 0.917).

### 3.3. mRNA Expression Levels of the ENTPD1, NT5E, and ADORA2A Genes in PBLs Obtained from Patients with MASLD and Healthy Donors

**Figure 3:**
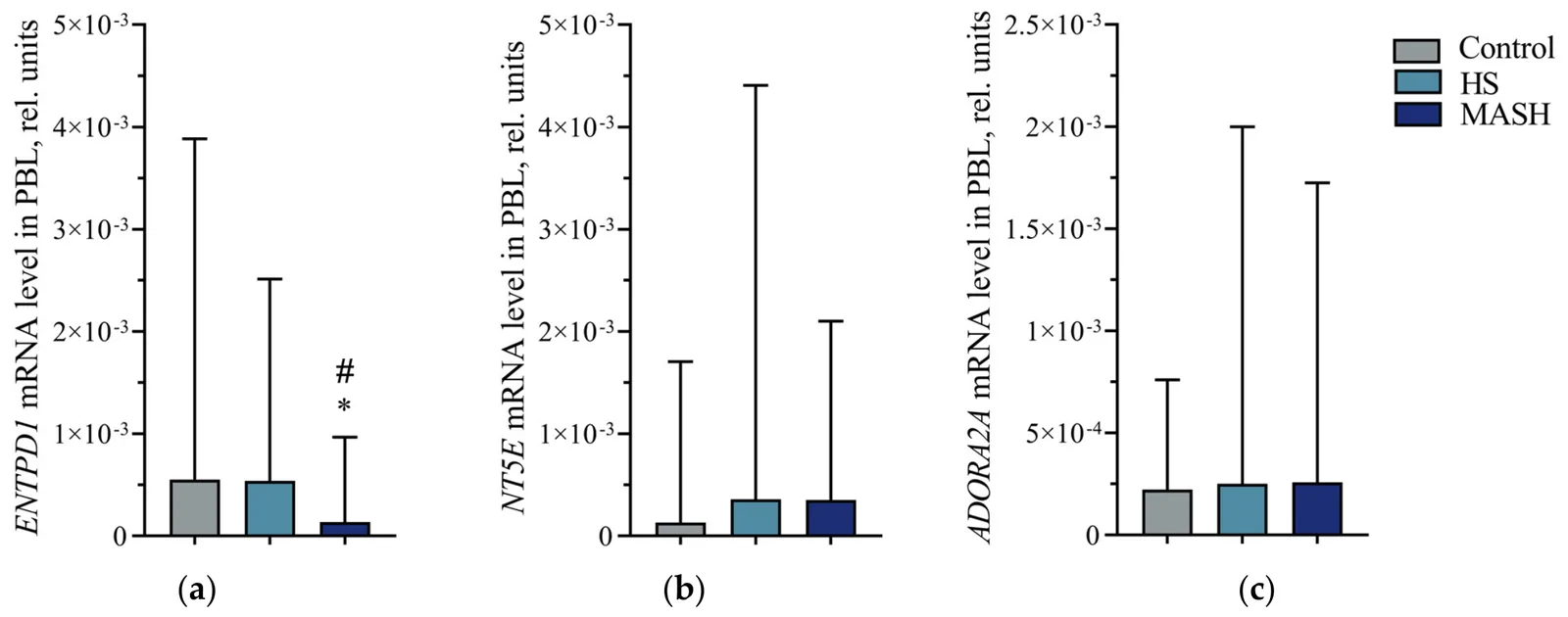
(**a**) mRNA levels of the *ENTPD1* gene in PBLs of patients with MASLD and healthy donors. (**b**) mRNA levels of the *NT5E* gene in PBLs of patients with MASLD and healthy donors. (**c**) mRNA levels of the *ADORA2A* gene in PBLs of patients with MASLD and healthy donors. *—Significant difference from the control group; #—significant difference from the HS group (according to the Mann–Whitney *U* test with Bonferroni correction). Medians and interquartile ranges are presented. *ENTPD1*—gene encoding ectonucleoside triphosphate diphosphohydrolase 1; *NT5E*—gene encoding ecto-5′-nucleotidase; *ADORA2A*—gene encoding adenosine A2A receptor; PBL—peripheral blood leukocytes; HS—hepatic steatosis; MASH—metabolic dysfunction-associated steatohepatitis.

According to the Kruskal–Wallis analysis of variance, the study groups differed in the *ENTPD1* gene mRNA level in PBLs (*H* = 9.269, *p* = 0.010). The *ENTPD1* gene mRNA level in MASH patients was lower than in healthy individuals and patients with simple hepatic steatosis (Figure 3a). The study groups did not differ in the mRNA levels of the *NT5E* and *ADORA2A* genes in PBLs (*H* = 3.859, *p* = 0.145; *H* = 1.347, *p* = 0.510, respectively) (Figure 3b,c, respectively).

### 3.4. Diagnostic Value of ADK Concentration and Relative mRNA Level of ENTPD1 Gene in PBLs for Differentiation of MASH from HS

**Figure 4:**
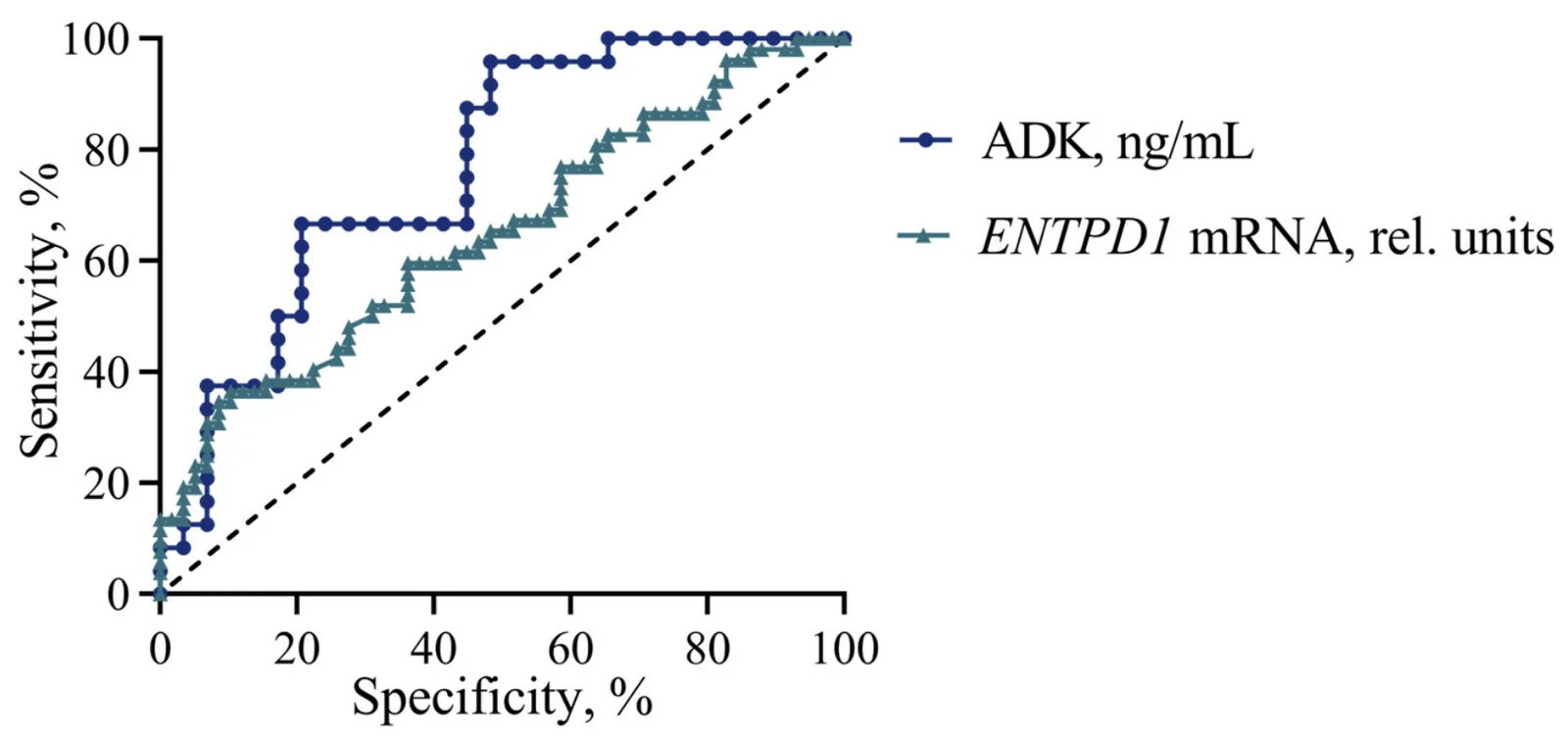
ROC curves of ADK concentration and relative level of the *ENTPD1* gene transcripts in PBLs of patients with MASH relative to HS. The dashed diagonal line represents chance-level classification, where the false positive rate (FPR) equals the true positive rate (TPR). *AUC* (ADK, ng/mL) = 0.764 (95% CI 0.637–0.892), *p* = 0.001. *AUC* (*ENTPD1* mRNA, rel. units) = 0.649 (95% CI 0.546–0.752), *p* = 0.007. ADK—adenosine kinase; *ENTPD1*—gene encoding ectonucleoside triphosphate diphosphohydrolase 1.

To assess the significance of ADK concentration and *ENTPD1* mRNA level in PBLs as diagnostic tests for distinguishing between MASLD forms, we constructed and analyzed ROC curves for these parameters in patients with MASH relative to patients with hepatic steatosis. According to the results of the ROC analysis, the ADK concentration and the mRNA level of the *ENTPD1* gene in PBLs of patients with MASH relative to HS have diagnostic value for differentiating MASH from HS, while the concentration of ADK in the blood has greater prognostic power in diagnosing MASH among patients with HS (Figure 4).

According to the maximum Youden index, the cutoff thresholds for detecting MASH among patients with HS are >25.67 ng/mL of ADK and <5.81 × 10^−5^ rel. units of *ENTPD1* mRNA in PBLs.

### 3.5. Content of CD14^+^ Monocytes and CD4^+^ T Helper Cells Expressing CD39 and CD73 in the Blood of Patients with MASLD

**Figure 5:**
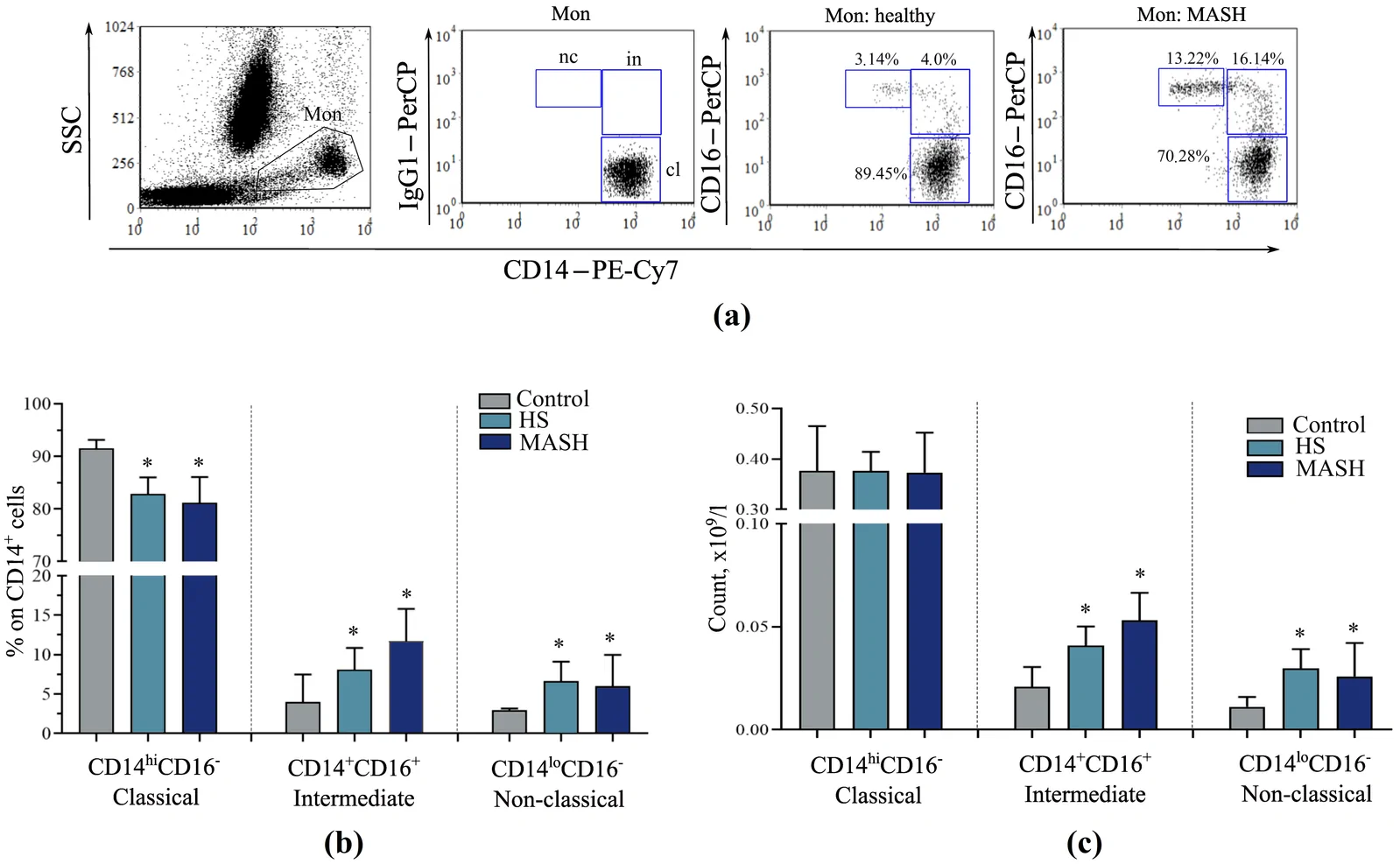
Distribution of monocyte subsets in peripheral blood in patients with HS, MASH, and healthy donors: (**a**) The gating strategy for monocyte populations based on anti-CD14-PE-Cy7 and anti-CD16-PerCP staining: cl—CD14^hi^CD16^−^ classical, in—CD14^+^CD16^+^ intermediate, and nc—CD14^lo^CD16^+^ non-classical monocytes. (**b**,**c**) Count of monocyte subsets. *—Significant difference from the control group (according to the Mann–Whitney *U* test with Bonferroni correction). Medians and interquartile ranges are presented.Mon—monocyte gate; HS—hepatic steatosis; MASH—metabolic dysfunction-associated steatohepatitis.

According to the clinical blood test, patients with MASLD generally did not differ in the content of peripheral lymphocytes and monocytes from the controls (Table 1). However, when analyzing monocyte subpopulations, it was noted that the studied groups statistically differed in the relative numbers of classical (*H* = 18.400, *p* < 0.001), intermediate (*H* = 14.800, *p* < 0.001), and non-classical (*H* = 19.120, *p* < 0.001) monocytes, as well as in the absolute numbers of intermediate (*H* = 13.500, *p* = 0.001) and non-classical (*H* = 20.140, *p* < 0.001) monocytes (Figure 5). In patients with MASLD, the relative abundance of classical monocytes was reduced compared with controls (Figure 5b), whereas the numbers of proinflammatory intermediate and non-classical monocytes in MASLD were increased (Figure 5b,c).

**Figure 6:**
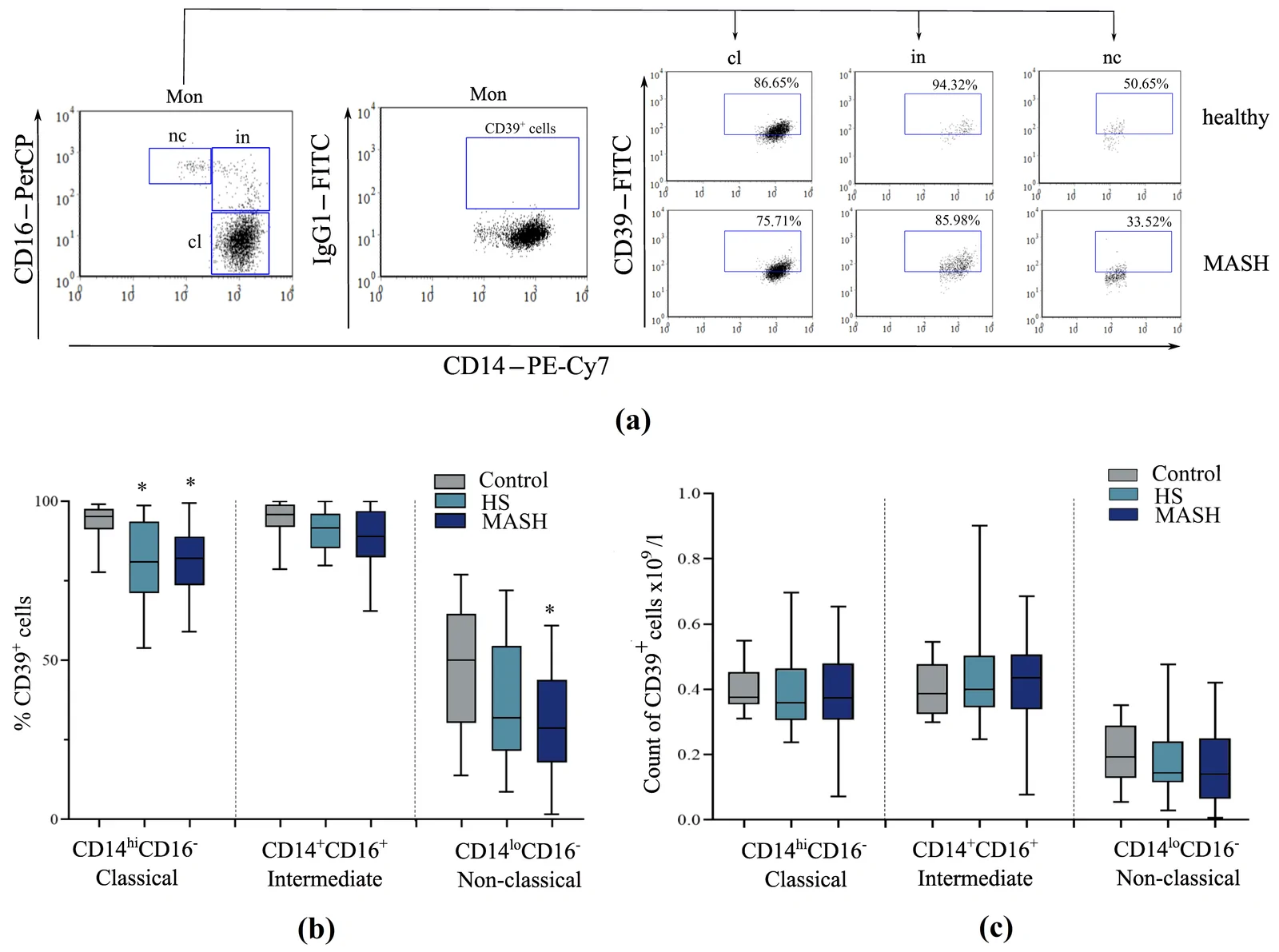
Distribution of CD39^+^ monocyte subsets in peripheral blood in patients with HS, MASH, and healthy donors: (**a**) Representative dot plots of the distribution of CD39^+^ cells among monocyte subsets in a patient with MASLD and a healthy donor. (**b**,**c**) Count of CD39^+^ monocyte subsets. *—Significant difference from the control group (according to the Mann–Whitney *U* test with Bonferroni correction). Medians, interquartile ranges, and minimum and maximum values are presented.Mon—monocyte gate; cl—CD14^hi^CD16^−^ classical, in—CD14^+^CD16^+^ intermediate, and nc—CD14^lo^CD16^+^ non-classical monocytes; HS—hepatic steatosis; MASH—metabolic dysfunction-associated steatohepatitis.

According to the Kruskal–Wallis analysis of variance, the study groups differed in the relative content of CD39-positive classical (*H* = 13.630, *p* = 0.001) and non-classical monocytes (*H* = 6.590, *p* = 0.037), but not in the absolute content (Figure 6c). In patients with HS, the level of CD39^+^ classical monocytes was reduced compared to the controls, and in patients with MASH, the levels of CD39^+^ classical and non-classical monocytes were reduced (Figure 6b). The obtained data are consistent with the results of the analysis of the mRNA level of the *ENTPD1* gene, which encodes CD39 (Figure 3a).

**Figure 7:**
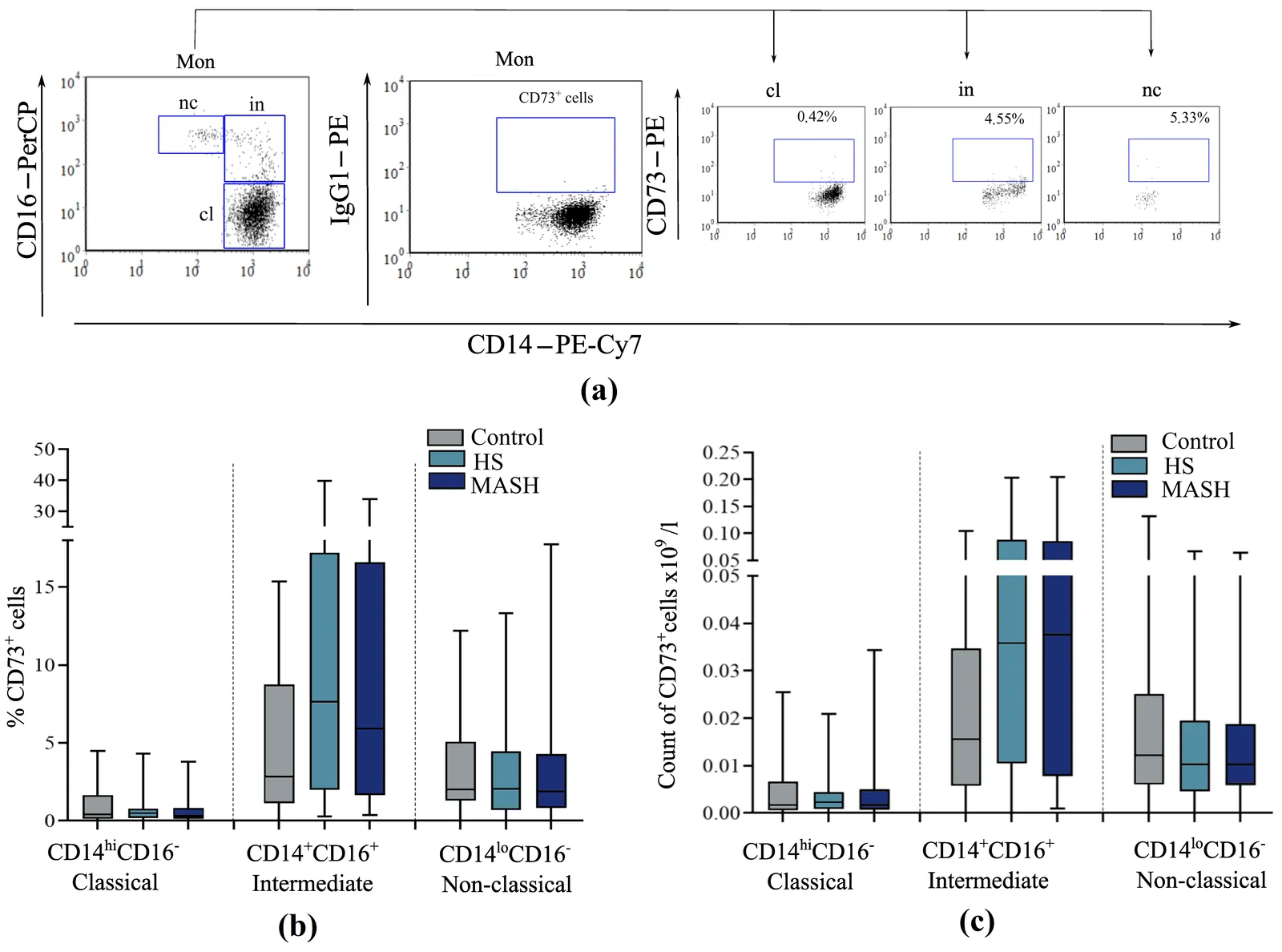
Distribution of CD73^+^ monocyte subsets in peripheral blood in patients with HS, MASH, and healthy donors: (**a**) Representative dot plots of the distribution of CD73^+^ cells among monocyte subsets in a healthy donor. (**b**,**c**) Count of CD73^+^ monocyte subsets. Medians, interquartile ranges, and minimum and maximum values are presented. Mon—monocyte gate; cl—CD14^hi^CD16^−^ classical, in—CD14^+^CD16^+^ intermediate, and nc—CD14^lo^CD16^+^ non-classical monocytes; HS—hepatic steatosis; MASH—metabolic dysfunction-associated steatohepatitis.

When analyzing the levels of CD73-expressing cells among monocyte subpopulations, no significant intergroup differences were found in the studied groups (Figure 7). It was noted that CD73 expression is not characteristic of classical and non-classical monocytes; a very small number of these monocytes were CD73-positive (in the control, for example, 1.39% (0.49–1.79%) and 5.48% (0.34–6.49%), respectively). More CD73^+^ cells were found in the subpopulation of intermediate monocytes (in the control, for example, 8.80% (6.61–15.55%)).

**Figure 8:**
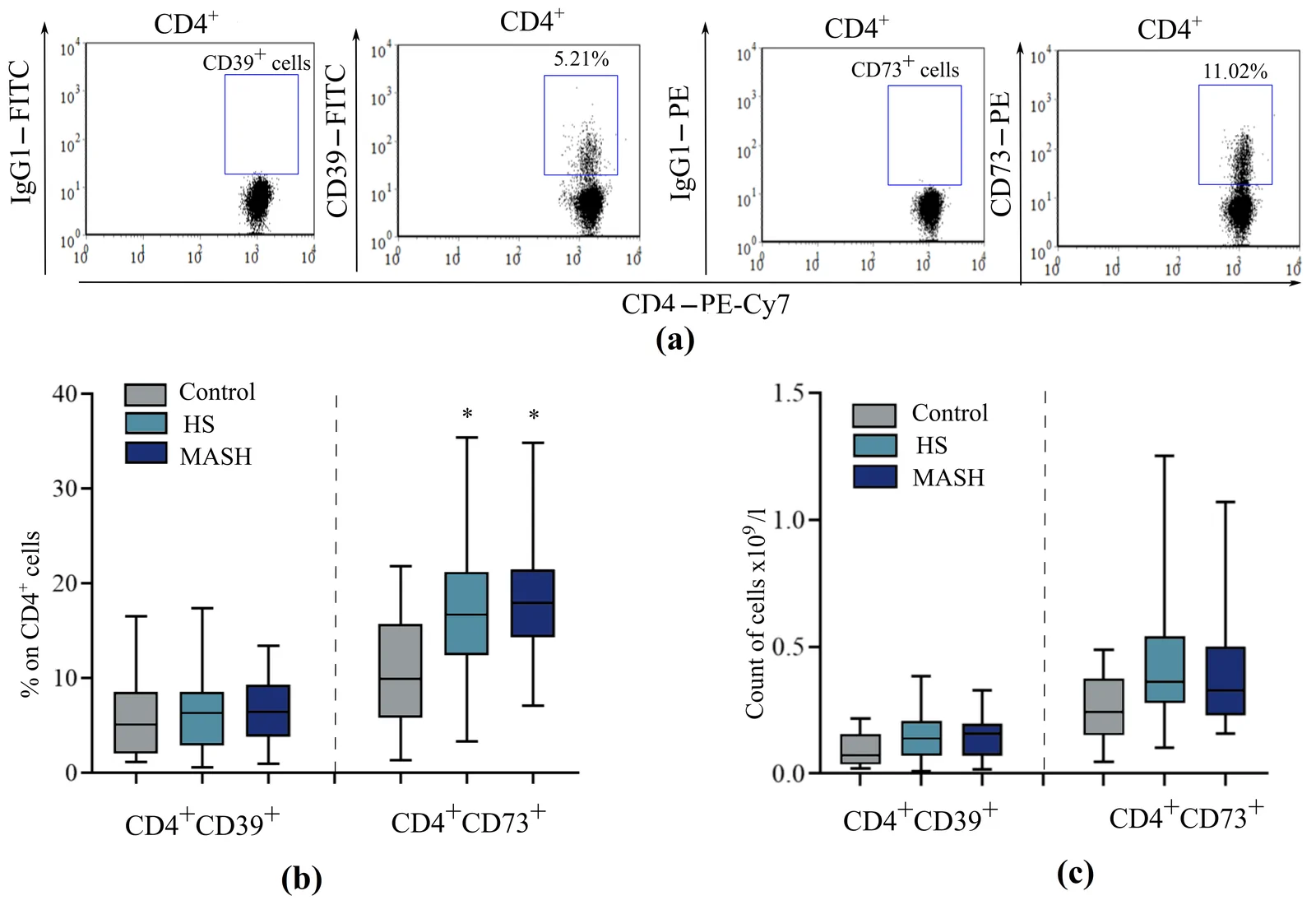
Distribution of CD39^+^ and CD73^+^ CD4^+^ Th cells in peripheral blood in patients with HS, MASH, and healthy donors: (**a**) Representative dot plots of the distribution of CD39^+^ and CD73^+^ cells among CD4^+^ T cells in a healthy donor. (**b**,**c**) Count of CD39^+^ and CD73^+^ CD4^+^ T cells. *—Significant difference from the control group (according to the Mann–Whitney *U* test with Bonferroni correction). Data are displayed as medians with interquartile ranges and minimum and maximum values.HS—hepatic steatosis; MASH—metabolic dysfunction-associated steatohepatitis.

Evaluation of peripheral CD4^+^ T cells revealed intergroup differences in the relative content of CD73-expressing cells (*H* = 15.180, *p* < 0.001). In patients with HS and MASH, the content of CD4^+^CD73^+^ T cells was similar and increased compared to controls (Figure 8). The level of CD39^+^CD4^+^ T cells in MASLD patients did not differ from that of healthy donors.

### 3.6. Concentrations of Proinflammatory (TNFα, IL-6, and IL-1β) and Anti-Inflammatory (IL10) Cytokines in Blood Plasma of Patients with MASLD and Healthy Donors

According to the Kruskal–Wallis analysis of variance, the study groups differed in the TNFα (*H* = 33.250, *p* < 0.001), IL-6 (*H* = 30.420, *p* < 0.001), IL-1β (*H* = 8.570, *p* = 0.014), and IL-10 (*H* = 14.270, *p* = 0.001) levels in the blood plasma. In patients with MASLD (HS and MASH), the levels of proinflammatory cytokines TNFα, IL-6, and IL-1β exceeded the control levels, whereas there were no significant differences between the forms of MASLD (Table 2). The level of the anti-inflammatory cytokine IL-10 in the blood plasma of patients with MASH was higher than in healthy individuals and patients with HS, while the concentration of IL-10 in HS was at the control level (Table 2).

### 3.7. Correlations Between ADK and mRNA Levels of Spliced Forms of the ADK Gene in PBLs and the Levels of Clinical Parameters and Cytokines

An assessment of the correlations between ADK content and mRNA levels of spliced forms of the *ADK* gene in PBLs with routine clinical parameters (Table 1), as well as the levels of the studied cytokines, was performed (Table 2). Table 3 presents the results of the Spearman correlation analysis for those parameters for which a correlation was found.

A positive correlation was found between the mRNA levels of the short and long isoforms of the *ADK* gene in PBLs in all study groups, as well as a positive correlation between the mRNA level of the gene encoding CD73 and the mRNA levels of the *ADK-S* and *ADK-L* isoforms. The transcript levels of the *ENTPD1* and *ADORA2A* genes positively correlated with the *ADK-S* mRNA level in the control group and with the *ADK-L* mRNA level in the HS and MASH groups. As for the level of ADK in PBL, positive correlations were established between this parameter and the level of *ENTPD1* mRNA in healthy individuals and between the ADK concentration in PBL and the TNFα level in the blood plasma of patients with MASH (Table 3). Furthermore, in MASH patients, a positive correlation was found between ADK levels and the levels of CD39-positive T helper cells and non-classical monocytes.

Thus, we have demonstrated for the first time that patients with MASLD with different clinical and morphological forms of this disease differ in the concentration of ADK and the relative mRNA level of the gene encoding CD39 in peripheral blood. In MASH, the concentration of ADK is higher, and the level of *ENTPD1* mRNA is lower than in simple hepatic steatosis, which has diagnostic significance for differentiating MASH from steatosis. Decreased levels of CD39^+^CD14^hi^CD16^−^ classical monocytes, but not CD4^+^CD39^+^ T cells, are likely associated with decreased *ENTPD1* gene mRNA levels in PBLs of patients with MASH. Analysis of correlations revealed that in healthy individuals, the level of ADK and the level of *ENTPD1* mRNA correlate positively with high strength according to the Chaddock scale; this correlation is absent in patients with MASLD. In patients with steatosis and MASH, the level of *ENTPD1* mRNA positively correlates with the level of mRNA of the long isoform of the *ADK* gene. In addition, in the blood of patients with MASH, the level of ADK positively correlates with the level of one of the key proinflammatory cytokines—TNFα.

## 4. Discussion

In this study, we demonstrated that the levels of adenosine kinase and mRNA of the *ENTPD1* gene, encoding CD39, differ depending on the form of MASLD. Patients with MASH had higher levels of ADK (Figure 1) and lower levels of *ENTPD1* gene transcripts (Figure 3a) in the PBL compared with healthy individuals and patients with MASLD with simple hepatic steatosis. According to our data, this has diagnostic value for identifying MASH among patients with HS (Figure 4).

In our analysis of the scientific literature, we did not find any data on the ADK content in PBL, but it is known that ADK is present in most tissues, with the highest amount in the liver [30,31]. There is evidence that the ADK level in the liver was increased in MASLD patients compared to patients without MASLD [35]. It should be noted that the authors of this work referred to the description of human liver samples presented in [43], which indicated that the authors used fixed liver sections of human subjects that are commercially available. This work did not present the clinical characteristics of the MASLD patients from whom the liver biopsies were obtained, and only severe hepatic steatosis was described in the tissue samples [43].

Evidence has accumulated on the significant role of ADK in the mechanisms associated with the pathogenesis of MASLD: lipotoxicity, insulin resistance, and inflammation. Thus, hepatic ADK content rises in mice with diet-induced obesity, steatosis, and systemic insulin resistance [35]. Conversely, hepatocyte ADK deficiency lowered hepatic fat accumulation and upregulated carnitine palmitoyltransferase 1a (*Cpt1a*) and peroxisome proliferator-activated receptor alpha (*Ppara*) genes, encoding molecules that determine the rate of fatty acid oxidation [35]. Hepatocyte-specific ADK disruption (Hep-ADK^+/−^) attenuated diet-induced steatosis, inflammation, and systemic insulin resistance in mice; moreover, under a methionine- and choline-deficient diet (MCD), these animals developed milder MASH than Hep-ADK^+/+^ controls. Opposite effects followed hepatocyte-specific ADK overexpression, which augmented body weight, obesity, steatosis, and liver inflammation relative to controls [35]. Accumulating evidence implicates elevated ADK in insulin resistance. β-cell ADK ablation improved glucose tolerance and β-cell function in mice while promoting β-cell proliferation and islet expansion [36]. In HFD-fed obese mice, endothelial ADK was markedly upregulated; deficiency of this enzyme improved glucose tolerance and insulin sensitivity and reduced hepatic steatosis, adipose inflammation, and obesity, effects linked to greater NO production via enhanced expression and activation of nitric oxide synthase 3 [34].

MASLD development involves inflammatory activation, fibrosis, and apoptosis [44,45]. In HS, low-grade chronic inflammation arises, triggered in part by endoplasmic reticulum stress in adipocytes amid an expanded free fatty acid pool. Inflammation is initiated and maintained by dysfunctional adipocytes secreting inflammatory adipocytokines, infiltration of immune cells producing cytokines and chemokines, and activated adipose tissue macrophages, which subsequently leads to the development of insulin resistance, the onset of hepatocellular ballooning degeneration, and lobular and portal inflammation [46,47]. Inflammatory processes in MASLD patients are initiated already at the stage of simple steatosis, which is confirmed by data on the increase in proinflammatory cytokines in the blood of patients in the HS and MASH groups compared to the control group (Table 2). Furthermore, the presence of inflammation in MASLD is indicated by elevated levels of pro-inflammatory CD16^+^ monocytes (intermediate and non-classical), a finding demonstrated in our study as well as in the work of other authors [48,49].

The observed increase in ADK levels in PBL in patients with MASH compared to those with simple hepatic steatosis may be related to the pathological role of excess ADK in the liver in the progression of steatosis, liver inflammation, and insulin resistance. This hypothesis may be supported, at least in part and indirectly, by the observed positive correlation (classified as strong on the Chaddock scale) between ADK concentration in PBL and plasma levels of the proinflammatory cytokine TNFα in patients with MASH (Table 3). However, it should be noted that both of these markers may reflect inflammation independently of one another.

At the same time, it is known that acute or constitutive deficiency of ADK due to mutations/knockout of the *ADK* gene can lead to severe metabolic disorders, causing a decrease in ATP reserves and accumulation of S-adenosylhomocysteine and adenosine, which leads to liver dysfunction, including steatosis, inflammation, and carbohydrate metabolism disorders [31,32,33]. It can be concluded that both deficiency and excess of ADK negatively affect liver homeostasis, which is likely due to the extremely important role of this enzyme in the regulation of metabolism. Within the cell, ADK phosphorylates adenosine to AMP and thus governs intracellular and extracellular levels of the nucleoside, which are balanced by equilibrative nucleoside transporters. In this way, the enzyme can modulate adenosine receptor activation in purinergic signaling [30]. ADK also contributes to transmethylation by regulating the thermodynamic equilibrium of S-adenosyl-L-homocysteine (SAH) hydrolysis to adenosine and homocysteine, catalyzed by S-adenosylhomocysteine hydrolase (SAHH). Because SAH competitively inhibits DNA methyltransferases, its concentration is critical. The SAHH reaction is shifted toward SAH formation and can proceed only when adenosine remains low and is efficiently removed by ADK—a condition essential for methyl-group flux along this pathway [29,50].

Recent data indicate that ADK, by maintaining metabolic adenosine clearance, protects hepatocytes against TNFα-induced apoptosis [51]. Lowering adenosine levels, in turn, activates the methionine cycle and transmethylation reactions. The authors showed that this enzyme acts as an endogenous suppressor of receptor-interacting serine/threonine protein kinase 1 (RIPK1), ensuring constitutive symmetric demethylation of R606 in the death domain of this kinase, catalyzed by protein arginine methyltransferase 5; this prevents kinase activation and blocks cell death. Knockout of the *Adk* gene in hepatocytes led to spontaneous liver injury in mice in the absence of stress factors. ADK deficiency also increased the expression of proinflammatory cytokines (including TNFα, IL-6, and IL-1β) in the liver, which was prevented by inactivation of RIPK1 [51]. In mice, ADK deficiency in the liver has been shown to aggravate acetaminophen-induced acute liver injury (ALI), whereas ADK overexpression protects against ALI by activating autophagy through the AMPK-mTOR and ADORA1-Akt-mTOR signaling pathways [52]. The presented data indicate both the pathological role of excess hepatic ADK in the progression of steatosis, liver inflammation, and insulin resistance—processes underlying the progression of MASLD—and the activation of protective mechanisms involving ADK. The elevated ADK levels in PBLs observed in our study in cases of MASH, compared to simple hepatic steatosis, are likely linked to the progression of MASLD; however, this should be the subject of further research due to the limitations of our study.

The method for determining ADK concentrations in PBLs used in our study involves determining the total ADK content without taking into account the different cellular localizations of the two isoforms of this enzyme. The long form (ADK-L) is known to be located in the nucleus, while the short form (ADK-S) is found in the cytoplasm [28]. Moreover, the isoforms may perform different functions: ADK-L is involved in nuclear transmethylation reactions, while ADK-S is involved in adenosine receptor-dependent mechanisms and, possibly, in cytosolic transmethylation functions [29]. In our study, we assessed the relative mRNA levels of the *ADK-S* and *ADK-L* genes in the PBLs of patients with MASLD (HS and MASH) and healthy donors and found no intergroup differences (Figure 2). An assessment of the relative mRNA levels of the long and short isoforms of the *ADK* gene in PBLs revealed that the ratio of the relative mRNA level of *ADK-L* to that of *ADK-S* exceeds 1. Our data focus on mRNA levels and do not directly reflect the abundance of protein isoforms. According to other researchers, immunoblotting analysis demonstrated higher levels of ADK-L expression compared to ADK-S in hepatocytes [51].

The level of adenosine in the cell is regulated not only by the rate of its utilization but also by the processes of its generation from ATP, in which CD39 and CD73 are the key enzymes. We showed that in the PBLs of patients with MASH, along with an increased level of ADK, the level of *ENTPD1* mRNA, encoding CD39, was reduced compared to patients with HS and healthy individuals (Figure 3a). Moreover, the level of *ENTPD1* gene transcripts positively correlated with the level of *ADK-S* mRNA and the concentration of ADK in PBLs in healthy individuals and with the level of *ADK-L* mRNA in the HS and MASH groups (Table 3), indicating a complex relationship between the regulation of the content of these molecules in PBLs. CD39 is known to be present on the surface of many immune cells [14]. In this study, we demonstrated that patients with MASLD are characterized by reduced levels of CD39-expressing monocytes, but not CD4^+^CD39^+^ Th cells (Figure 6 and Figure 8). Moreover, in MASLD, a reduced level of CD39^+^ cells compared to healthy donors was observed for all monocyte subsets, both for CD14^hi^CD16^−^ classical and for proinflammatory CD14^+^CD16^+^ intermediate and CD14^lo^CD16^+^ non-classical monocytes. Furthermore, the study revealed positive correlations between the concentration of ADK, the level of CD4^+^CD39^+^ Th cells, and the level of CD39^+^CD14^lo^CD16^+^ non-classical monocytes in patients with MASH (Table 3). The CD4^+^ Th cell population is heterogeneous. In our previous study [53], we demonstrated that the level of immunosuppressive CD4^+^CD39^+^ regulatory T cells (Treg cells) in the blood of patients with MASH was higher than in healthy donors. Activation of CD39 expression on other immune cell populations is likely associated with a deficiency of this ectonucleotidase in MASH or with the activation of immunosuppressive mechanisms. CD39 expression is important for the immunosuppressive function of Treg cells [54]. In MASH, a positive correlation between ADK concentration and the abundance of CD39^+^CD14^lo^CD16^+^ non-classical monocytes may likewise indicate activated adenosine metabolism.

Evidence has accumulated suggesting a pathological role for CD39 deficiency in liver injury. CD39 deletion in experimental models has been shown to cause insulin resistance and exacerbate liver inflammation. CD39 deficiency leads to activation of the AMP-activated protein kinase and mTOR pathways and increased ATP levels, which stimulates hepatocyte proliferation, suppresses autophagy, and disrupts glycolytic metabolism, leading to malignant transformation [18]. Furthermore, CD39 deficiency has been shown to play a role in bile duct injury and fibrosis through the recruitment of cytotoxic T cells [19]. Multiple studies confirm that CD39 is central to balancing effector and regulatory cells. Normal or elevated levels and activity of this enzyme produce beneficial immunological effects, whereas impaired expression and function probably disrupt this equilibrium, sustaining and advancing tissue damage [20]. CD39 mRNA-based therapy has been shown to be effective in regulating local inflammation in mice [21]. Our study demonstrated for the first time that patients with MASH exhibit lower levels of *ENTPD1* mRNA in peripheral blood leukocytes compared to those with simple hepatic steatosis.

In our study, no intergroup differences in *NT5E* mRNA levels were found in PBL (Figure 3b); a positive correlation was found between *NT5E* levels and *ADK-S* and *ADK-L* mRNA levels in all study groups (Table 3). This correlation is expected, since CD73 nucleotidase is an enzyme that dephosphorylates AMP to adenosine via both the classical pathway (CD39/CD73) and the non-classical pathway (CD38/CD203a/CD73) [55]. When analyzing CD73^+^ cells, no intergroup differences in the levels of CD73-positive monocytes were found in the study groups. In MASLD (HS and MASH), the level of CD4^+^CD73^+^ Th cells was higher than in the control group (Figure 8). The increased number of CD4^+^CD73^+^ T cells is likely associated with the high content of CD4^+^ T cells themselves in MASLD [8]. Independently of its enzymatic activity, CD73 delivers a costimulatory signal for lymphocyte activation and mediates their adhesion to the endothelium [56]. Like CD39, this ectonucleotidase exerts anti-inflammatory effects. In a diet-induced MASLD model, knockout of the gene encoding CD73 led to disease progression and reduced the viability and activity of intrahepatic Tregs. Adoptively transferred wild-type Tregs showed greater suppressive activity and protected against hepatic inflammation in MASLD than those from CD73-knockout donors. Degradation of AMP to adenosine enhances Treg function and blocks DR5-TRAIL-mediated cell death signals [22]. Nevertheless, the enzyme may also be pathogenic in liver disease: its deficiency protected mice from CCl4- and thioacetamide-induced fibrosis, and hepatic collagen content after fibrosis induction was markedly lower in knockout animals than in wild-type controls [23]. Recent evidence further implicates CD73 in fibrogenesis via activation and proliferation of hepatic stellate cells [24].

It is known that the deficiency of the A2A adenosine receptor, both in macrophages and hepatocytes, is accompanied by an increase in the severity of MASLD, probably due to increased inflammation and lipogenesis [16]. A2AR deficiency markedly elevated sterol regulatory element-binding protein 1c (SREBP1c) abundance in the livers of fasted mice and in nutrient-deprived hepatocytes, an effect contributing to the antisteatotic action of A2AR [16]. It was also shown that A2AR-disrupted mice treated with MCD exhibited an increase in liver weight and an increase in the severity of liver steatosis compared to control mice also treated with MCD [17]. Given the evidence for a possible protective role of A2AR in MASLD progression, in our study, we also assessed the relative mRNA level of the *ADORA2A* gene in PBLs of patients with HS, MASH, and healthy individuals and found no intergroup differences (Figure 3c).

## 5. Conclusions

Thus, we have demonstrated for the first time that the level of ADK in peripheral leukocytes may be associated with the presence and activity of inflammation in MASLD, most often originating from inflammation in the liver parenchyma. An increase in ADK levels in PBL was detected in MASH (relative to simple hepatic steatosis and normal values). This may be related both to the pathological role of excess ADK in the progression of steatosis, liver inflammation, and insulin resistance, which underlie the progression of MASLD, and to the activation of protective mechanisms involving ADK. It was also shown that the relative level of *ENTPD1* gene transcripts in PBL was reduced in MASH, which is most likely associated with low levels of CD39^+^ monocytes. This may contribute to the progression of MASLD. To the best of our knowledge, this is the first study to report that ADK and *ENTPD1* mRNA levels in PBL may help distinguish between MASLD forms, particularly in identifying individuals with MASH among patients with HS. These findings suggest a potential discriminative ability of these markers within the studied cohort, warranting further validation in independent populations. These data may help address the challenge of differentiating MASLD forms using a minimally invasive approach and may expand our understanding of the mechanisms underlying MASLD progression, ultimately contributing to improved treatment efficacy.

## 6. Limitations

This study did not measure hepatic ADK levels, nor did it assess the activity of this enzyme or adenosine levels. It is a cross-sectional study comparing existing disease states. The potential association between elevated ADK levels and reduced *ENTPD1* mRNA levels in PBLs and the progression of MASLD warrants further investigation, given the study’s limitations. The threshold values and *AUC* estimates derived from the ROC analysis are specific to this cohort and require external validation in larger, independent cohorts.

## Figures and Tables

**Table 1:** Clinical and laboratory characteristics of the study groups.

Characteristic	Control	HS	MASH
*n*	42	50	48
Age, years	45.00 (35.00–51.00)	48.00 (39.00–53.00)	47.09 (39.00–54.00)
Male/Female (*n*)	20/22	26/24	24/24
ALT, units/L	14.45 (11.35–19.58)	23.80 (17.70–31.65) *	65.40 (53.85–96.80) *#
AST, units/L	19.90 (15.90–22.50)	22.05 (19.50–26.93)	46.50 (38.20–66.30) *#
Total bilirubin, mmol/L	10.85 (8.45–17.08)	15.00 (10.05–20.60)	13.25 (11.33–20.45)
ALP, units/L	90.35 (67.15–118.66)	115.00 (80.40–140.80)	144.50 (74.88–164.50) *
GGT, units/L	15.00 (11.00–23.00)	33.85 (25.40–45.30) *	62.10 (25.60–95.30) *
Total protein, g/L	73.55 (69.95–75.25)	72.05 (66.90–77.75)	74.40 (68.10–78.50)
CRP, mg/L	0.50 (0.20–2.05)	2.55 (1.23–3.63)	2.80 (1.03–4.37) *
ESR, mm/h	6.00 (3.00–12.50)	6.00 (4.00–14.65)	10.00 (5.00–20.00)
Leukocytes, ×10^9^/L	5.89 (5.31–7.14)	6.42 (5.65–7.87)	6.45 (5.310–7.74)
Lymphocytes, ×10^9^/L	2.18 (1.67–2.48)	2.21 (1.91–2.78)	2.03 (1.64–2.70)
Monocytes, ×10^9^/L	0.40 (0.34–0.45)	0.46 (0.40–0.55)	0.48 (0.39–0.56)
Glucose, mmol/L	5.10 (4.87–5.52)	5.66 (5.09–5.75)	5.60 (5.16–6.00)
TC, mmol/L	4.97 (4.47–5.80)	4.94 (4.42–5.90)	5.65 (4.82–6.55) *#
HDL, mmol/L	1.30 (1.18–1.53)	1.12 (0.98–1.28) *	1.02 (0.67–1.13) *#
LDL, mmol/L	2.61 (2.35–3.01)	3.11 (3.00–3.40) *	3.39 (3.13–4.16) *#
Triglycerides, mmol/L	1.01 (0.74–1.98)	2.11 (1.37–2.79) *	1.96 (1.49–2.93) *
BMI, kg/m^2^	24.00 (20.51–24.90)	33.70 (30.30–35.51) *	35.68 (31.24–36.42) *
FIB-4	0.82 (0.63–0.88)	1.20 (0.88–2.00)	1.24 (0.98–2.01) *

HS—Hepatic steatosis; MASH—metabolic dysfunction-associated steatohepatitis; ALT—alanine aminotransferase; AST—aspartate aminotransferase; ALP—alkaline phosphatase; GGT—gamma-glutamyl transpeptidase; CRP—C-reactive protein; ESR—erythrocyte sedimentation rate; TC—total cholesterol; HDL—high-density lipoprotein; LDL—low-density lipoprotein; BMI—body mass index. Data are presented as medians, with lower and upper quartile values in parentheses. *—Significant difference from the control group; #—significant difference from the HS group (according to the Mann–Whitney *U* test with Bonferroni correction).

**Table 2:** Plasma cytokine levels in healthy individuals and patients with MASLD.

Characteristic	Control (*n* = 30)	HS (*n* = 43)	MASH (*n* = 46)
TNFα, pg/mL	3.550 (2.828–4.200)	5.490 (3.830–6.740) *	5.900 (4.518–7.095) *
IL-6, pg/mL	1.995 (1.528–2.510)	3.770 (2.868–5.688) *	3.645 (2.443–5.498) *
IL-1β, pg/mL	2.460 (1.300–3.400)	2.930 (2.328–4.230) *	3.265 (2.220–4.215) *
IL-10, pg/mL	6.325 (4.848–8.503)	7.000 (4.050–10.320)	9.975 (6.833–14.180) *^#^

Data are presented as medians, with lower and upper quartile values in parentheses. *—Significant difference from the control group; #—significant difference from the HS group (according to the Mann–Whitney *U* test with Bonferroni correction).

**Table 3:** Spearman correlation coefficients (*r*) of the ADK level in the blood, as well as the mRNA levels of spliced forms of the *ADK* gene in the PBL, with the level of the studied blood parameters in MASLD patients and healthy people (at *p* < 0.05).

Characteristic	Control			HS			MASH		
	ADK	*ADK-S* mRNA	*ADK-L* mRNA	ADK	*ADK-S* mRNA	*ADK-L* mRNA	ADK	*ADK-S* mRNA	*ADK-L* mRNA
GGT		0.50							
*ADK-S* mRNA			0.59			0.49			0.40
*ADK-L* mRNA		0.59			0.49			0.40	
*ENTPD1* mRNA	0.75	0.48				0.64			0.42
*NT5E* mRNA		0.55	0.93		0.56	0.85		0.43	0.78
*ADORA2A* mRNA		0.59				0.74			0.65
TNFα							0.52		
CD4^+^CD39^+^, % on CD4^+^ T cells							0.51		
CD14^+^CD39^+^, % on CD14^+^CD16^+^ non-classical monocytes							0.53		

## Data Availability

The original contributions presented in this study are included in the article. Further inquiries can be directed to the corresponding author.

## Abbreviations

The following abbreviations are used in this manuscript:

MASLD	Metabolic dysfunction-associated steatotic liver disease
NAFLD	Non-alcoholic fatty liver disease
HS	Hepatic steatosis
MASH	Metabolic dysfunction-associated steatohepatitis
A2AR	Adenosine A2A receptor
ATP	Adenosine triphosphate
ENTPD1	Ectonucleoside triphosphate diphosphohydrolase 1
ADP	Adenosine diphosphate
AMP	5′-Adenosine monophosphate
NT5E	Ecto-5′-nucleotidase
ADK	Adenosine kinase
ADK-L	Long isoform of the adenosine kinase
ADK-S	Short isoform of the adenosine kinase
HFD	High-fat diet
PBL	Peripheral blood leukocytes
TNFα	Tumor necrosis factor alpha
IL-6	Interleukin 6
IL-1β	Interleukin 1 beta
IL-10	Interleukin 10
Th	T helper cells
ALT	Alanine aminotransferase
AST	Aspartate aminotransferase
ALP	Alkaline phosphatase
GGT	Gamma-glutamyl transpeptidase
CRP	C-reactive protein
ESR	Erythrocyte sedimentation rate
TC	Total cholesterol
HDL	High-density lipoprotein
LDL	Low-density lipoprotein
BMI	Body mass index
ARFI	Acoustic Radiation Force Impulse
HBV	Hepatitis B virus
HCV	Hepatitis C virus
EDTA	Ethylenediaminetetraacetic acid
ELISA	Enzyme-linked immunosorbent assay
cDNA	Complementary DNA
RT-PCR	Real-time polymerase chain reaction
ROC	Receiver operating characteristic
AUC	Area under the curve
Cpt1a	Carnitine palmitoyltransferase 1a
Ppara	Peroxisome proliferator-activated receptor alpha
MCD	Methionine- and choline-deficient diet
SAH	S-adenosyl-L-homocysteine
SAHH	S-adenosylhomocysteine hydrolase
RIPK1	Receptor-interacting serine/threonine protein kinase 1
ALI	Acetaminophen-induced acute liver injury
Treg cells	Regulatory T cells
SREBP1c	Sterol regulatory element-binding protein 1c

## References

[1] Younossi Z.M., Golabi P., Paik J.M., Henry A., Van Dongen C., Henry L. (2023). The Global Epidemiology of Nonalcoholic Fatty Liver Disease (NAFLD) and Nonalcoholic Steatohepatitis (NASH): A Systematic Review. Hepatology.

[2] Amini-Salehi E., Letafatkar N., Norouzi N., Joukar F., Habibi A., Javid M., Sattari N., Khorasani M., Farahmand A., Tavakoli S. (2024). Global Prevalence of Nonalcoholic Fatty Liver Disease: An Updated Review Meta-Analysis Comprising a Population of 78 Million from 38 Countries. Arch. Med. Res..

[3] Loomba R., Friedman S.L., Shulman G.I. (2021). Mechanisms and Disease Consequences of Nonalcoholic Fatty Liver Disease. Cell.

[4] Adams L.A., Ratziu V. (2015). Non-Alcoholic Fatty Liver–Perhaps Not so Benign. J. Hepatol..

[5] Ristic-Medic D., Bajerska J., Vucic V. (2022). Crosstalk between dietary patterns, obesity and nonalcoholic fatty liver disease. World J. Gastroenterol..

[6] Ratziu V., Charlotte F., Heurtier A., Gombert S., Giral P., Bruckert E., Grimaldi A., Capron F., Poynard T. (2005). Sampling Variability of Liver Biopsy in Nonalcoholic Fatty Liver Disease. Gastroenterology.

[7] Peiseler M., Schwabe R., Hampe J., Kubes P., Heikenwälder M., Tacke F. (2022). Immune Mechanisms Linking Metabolic Injury to Inflammation and Fibrosis in Fatty Liver Disease—Novel Insights into Cellular Communication Circuits. J. Hepatol..

[8] Diedrich T., Kummer S., Galante A., Drolz A., Schlicker V., Lohse A.W., Kluwe J., Eberhard J.M., Schulze Zur Wiesch J. (2020). Characterization of the Immune Cell Landscape of Patients with NAFLD. PLoS ONE.

[9] Barreby E., Chen P., Aouadi M. (2022). Macrophage Functional Diversity in NAFLD—More than Inflammation. Nat. Rev. Endocrinol..

[10] Williams H., Mack C., Baraz R., Marimuthu R., Naralashetty S., Li S., Medbury H. (2023). Monocyte Differentiation and Heterogeneity: Inter-Subset and Interindividual Differences. Int. J. Mol. Sci..

[11] Pasquini S., Contri C., Borea P.A., Vincenzi F., Varani K. (2021). Adenosine and Inflammation: Here, There and Everywhere. Int. J. Mol. Sci..

[12] Antonioli L., Fornai M., Blandizzi C., Pacher P., Haskó G. (2019). Adenosine Signaling and the Immune System: When a Lot Could Be Too Much. Immunol. Lett..

[13] Zhulai G., Oleinik E., Shibaev M., Ignatev K. (2022). Adenosine-Metabolizing Enzymes, Adenosine Kinase and Adenosine Deaminase, in Cancer. Biomolecules.

[14] Jain S., Jacobson K.A. (2021). Purinergic Signaling in Liver Pathophysiology. Front. Endocrinol..

[15] Wang S., Gao S., Zhou D., Qian X., Luan J., Lv X. (2021). The Role of the CD39–CD73–Adenosine Pathway in Liver Disease. J. Cell. Physiol..

[16] Cai Y., Li H., Liu M., Pei Y., Zheng J., Zhou J., Luo X., Huang W., Ma L., Yang Q. (2018). Disruption of Adenosine 2A Receptor Exacerbates NAFLD through Increasing Inflammatory Responses and SREBP1c Activity. Hepatology.

[17] Zhou J., Li H., Cai Y., Ma L., Matthews D., Lu B., Zhu B., Chen Y., Qian X., Xiao X. (2019). Mice Lacking Adenosine 2A Receptor Reveal Increased Severity of MCD-Induced NASH. J. Endocrinol..

[18] Sun X., Imai M., Nowak-Machen M., Guckelberger O., Enjyoji K., Wu Y., Khalpey Z., Berberat P., Munasinghe J., Robson S.C. (2011). Liver Damage and Systemic Inflammatory Responses Are Exacerbated by the Genetic Deletion of CD39 in Total Hepatic Ischemia. Purinergic Signal..

[19] Peng Z., Rothweiler S., Wei G., Ikenaga N., Liu S.B., Sverdlov D.Y., Vaid K.A., Longhi M.S., Kuang M., Robson S.C. (2017). The Ectonucleotidase ENTPD1/CD39 Limits Biliary Injury and Fibrosis in Mouse Models of Sclerosing Cholangitis. Hepatol. Commun..

[20] Savio L.E.B., Robson S.C., Longhi M.S. (2021). Ectonucleotidase Modulation of Lymphocyte Function in Gut and Liver. Front. Cell Dev. Biol..

[21] Philosof N.R., Walsh A.P.G., Xu B., Watson A.M.D., Ren S., Hann L., Nathaniel B., Nguyen A., Liu H., Shi P. (2026). CD39 mRNA Therapy Attenuates Localized Acute Inflammation: A Novel Anti-Inflammatory Strategy Using Cationic Nanoliposomes. Mol. Ther. Nucleic Acids.

[22] Jin H., Zhong X., Zhang C., Wu Y., Sun J., Wang X., Wang Z., Zhu J., Jiang Y., Du X. (2025). CD73 Promotes the Immunoregulatory Functions of Hepatic Tregs through Enzymatic and Nonenzymatic Pathways in MASLD Development. Mol. Metab..

[23] Peng Z., Fernandez P., Wilder T., Yee H., Chiriboga L., Chan E.S.L., Cronstein B.N. (2008). Ecto-5′-nucleotidase (CD73) -mediated Extracellular Adenosine Production Plays a Critical Role in Hepatic Fibrosis. FASEB J..

[24] Yang L., Gao Z.-W., Wu X.-N., Liu C., Zhang J., Zhang H.-Z., Dong K. (2023). CD73 Blockade Alleviated Hepatic Fibrosis via Inhibiting Hepatic Stellate CellsProliferation and Activation. Curr. Mol. Pharmacol..

[25] Park J., Gupta R.S. (2008). Adenosine Kinase and Ribokinase—The RK Family of Proteins. Cell. Mol. Life Sci..

[26] Peter-Okaka U., Boison D. (2025). Adenosine Kinase: An Epigenetic Modulator and Drug Target. J. Inherit. Metab. Dis..

[27] Cui X.A., Agarwal T., Singh B., Gupta R.S. (2011). Molecular Characterization of Chinese Hamster Cells Mutants Affected in Adenosine Kinase and Showing Novel Genetic and Biochemical Characteristics. BMC Biochem..

[28] Cui X.A., Singh B., Park J., Gupta R.S. (2009). Subcellular Localization of Adenosine Kinase in Mammalian Cells: The Long Isoform of AdK Is Localized in the Nucleus. Biochem. Biophys. Res. Commun..

[29] Murugan M., Fedele D., Millner D., Alharfoush E., Vegunta G., Boison D. (2021). Adenosine Kinase: An Epigenetic Modulator in Development and Disease. Neurochem. Int..

[30] Boison D., Scheurer L., Zumsteg V., Rülicke T., Litynski P., Fowler B., Brandner S., Mohler H. (2002). Neonatal Hepatic Steatosis by Disruption of the Adenosine Kinase Gene. Proc. Natl. Acad. Sci. USA.

[31] Bjursell M.K., Blom H.J., Cayuela J.A., Engvall M.L., Lesko N., Balasubramaniam S., Brandberg G., Halldin M., Falkenberg M., Jakobs C. (2011). Adenosine Kinase Deficiency Disrupts the Methionine Cycle and Causes Hypermethioninemia, Encephalopathy, and Abnormal Liver Function. Am. J. Hum. Genet..

[32] Becker P.-H., Demir Z., Mozer Glassberg Y., Sevin C., Habes D., Imbard A., Mussini C., Rozenfeld Bar Lev M., Davit-Spraul A., Benoist J.-F. (2021). Adenosine Kinase Deficiency: Three New Cases and Diagnostic Value of Hypermethioninemia. Mol. Genet. Metab..

[33] Triono A., Iskandar K., Nurani N., Hidayati I.S., Nugrahanto A.P., Mooiindie K.H., Herini E.S. (2026). ADK Deficiency without Hypermethioninemia Presenting as Intractable Epilepsy: A Rare Neurometabolic Case and Literature Review. Neurogenetics.

[34] Xu J., Yang Q., Zhang X., Liu Z., Cao Y., Wang L., Zhou Y., Zeng X., Ma Q., Xu Y. (2019). Endothelial Adenosine Kinase Deficiency Ameliorates Diet-Induced Insulin Resistance. J. Endocrinol..

[35] Li H., Zheng J., Xu Q., Yang Y., Zhou J., Guo X., Cai Y., Cai J.J., Xie L., Awika J. (2023). Hepatocyte Adenosine Kinase Promotes Excessive Fat Deposition and Liver Inflammation. Gastroenterology.

[36] Osman M.A.A., Sun Y.J., Li R.J., Lin H., Zeng D.M., Chen X.Y., He D., Feng H.W., Yang Z., Wang J. (2019). Deletion of pancreatic β-cell adenosine kinase improves glucose homeostasis in young mice and ameliorates streptozotocin-induced hyperglycaemia. J. Cell. Mol. Med..

[37] Brunt E.M., Janney C.G., Di Bisceglie A.M., Neuschwander-Tetri B.A., Bacon B.R. (1999). Nonalcoholic Steatohepatitis: A Proposal for Grading and Staging the Histological Lesions. Am. J. Gastroenterol..

[38] Sterling R.K., Lissen E., Clumeck N., Sola R., Correa M.C., Montaner J., Sulkowski M.S., Torriani F.J., Dieterich D.T., Thomas D.L. (2006). Development of a Simple Noninvasive Index to Predict Significant Fibrosis in Patients with HIV/HCV Coinfection. Hepatology.

[39] Shah A.G., Lydecker A., Murray K., Tetri B.N., Contos M.J., Sanyal A.J. (2009). Comparison of Noninvasive Markers of Fibrosis in Patients with Nonalcoholic Fatty Liver Disease. Clin. Gastroenterol. Hepatol..

[40] Livak K.J., Schmittgen T.D. (2001). Analysis of Relative Gene Expression Data Using Real-Time Quantitative PCR and the 2^−ΔΔCT^ Method. Methods.

[41] Youden W.J. (1950). Index for Rating Diagnostic Tests. Cancer.

[42] Zweig M.H., Campbell G. (1993). Receiver-Operating Characteristic (ROC) Plots: A Fundamental Evaluation Tool in Clinical Medicine. Clin. Chem..

[43] Luo X., Li H., Ma L., Zhou J., Guo X., Woo S.-L., Pei Y., Knight L.R., Deveau M., Chen Y. (2018). Expression of STING Is Increased in Liver Tissues from Patients with NAFLD and Promotes Macrophage-Mediated Hepatic Inflammation and Fibrosis in Mice. Gastroenterology.

[44] Powell E.E., Wong V.W.-S., Rinella M. (2021). Non-Alcoholic Fatty Liver Disease. Lancet.

[45] Kurbatova I.V., Topchieva L.V., Dudanova O.P., Shipovskaya A.A. (2024). Role of MMP-2 and MMP-9 in the Relationship between Inflammation, Fibrosis, and Apoptosis during Progression of Non-Alcoholic Fatty Liver Disease and Diagnostic Significance of Plasma Levels of Their Active Forms. Biochem. Mosc..

[46] Kawai T., Autieri M.V., Scalia R. (2021). Adipose Tissue Inflammation and Metabolic Dysfunction in Obesity. Am. J. Physiol. Cell Physiol..

[47] Li H., Meng Y., He S., Tan X., Zhang Y., Zhang X., Wang L., Zheng W. (2022). Macrophages, Chronic Inflammation, and Insulin Resistance. Cells.

[48] Zhang J., Chen W., Fang L., Li Q., Zhang X., Zhang H., Guan Q., Zhao R., Yang C., Jing F. (2018). Increased Intermediate Monocyte Fraction in Peripheral Blood Is Associated with Nonalcoholic Fatty Liver Disease. Wien. Klin. Wochenschr..

[49] Gadd V.L., Patel P.J., Jose S., Horsfall L., Powell E.E., Irvine K.M. (2016). Altered Peripheral Blood Monocyte Phenotype and Function in Chronic Liver Disease: Implications for Hepatic Recruitment and Systemic Inflammation. PLoS ONE.

[50] Boison D. (2013). Adenosine Kinase: Exploitation for Therapeutic Gain. Pharmacol. Rev..

[51] Liu R., Wang G., Jiang Z., Zou T., Wang C., Wang W., Cai M., Zhang S., Wang G., Cao H. (2026). Adenosine Metabolic Clearance Maintains Liver Homeostasis by Licensing Arginine Methylation of RIPK1. J. Exp. Med..

[52] Zhang C., Liu X., Liu X., Hua R., Liu H., Ma J., Zou D., Wang G., Yuan Q., Wang B. (2024). Adenosine Kinase Protects against Acetaminophen-Induced Acute Liver Injury by Activating Autophagy in Hepatocytes. Cell. Biol. Toxicol..

[53] Zhulai G., Kurbatova I., Dudaniva O. (2025). Blood Expression of CD39 and CD73 Ectonucleotidases in Patients with Various Forms of Metabolic-Associated Fatty Liver Disease. Bull. Russ. State Med. Univ..

[54] Timperi E., Barnaba V. (2021). CD39 Regulation and Functions in T Cells. Int. J. Mol. Sci..

[55] Ferretti E., Horenstein A.L., Canzonetta C., Costa F., Morandi F. (2019). Canonical and Non-Canonical Adenosinergic Pathways. Immunol. Lett..

[56] Schneider E., Rissiek A., Winzer R., Puig B., Rissiek B., Haag F., Mittrücker H.-W., Magnus T., Tolosa E. (2019). Generation and Function of Non-Cell-Bound CD73 in Inflammation. Front. Immunol..

